# Isolation, characterization and antimicrobial resistance of *Yersinia enterocolitica* from Polish cattle and their carcasses

**DOI:** 10.1186/s12917-023-03700-6

**Published:** 2023-09-05

**Authors:** Piotr Łada, Klaudia Kończyk-Kmiecik, Agata Bancerz-Kisiel

**Affiliations:** Department of Epizootiology, Faculty of Veterinary Medicine, University of Warmia and Mazury, Oczapowskiego 2 St, 10-719 Olsztyn, Poland

**Keywords:** *Yersinia enterocolitica*, Cattle, Cold-stored beef carcasses, Prevalence, Bioserotyping, Molecular characterization, Antimicrobial susceptibility, Multidrug resistance

## Abstract

**Background:**

*Yersinia enterocolitica* is a heterogeneous bacterial species that has been divided into six biotypes and more than 70 serotypes. Each year, the European Food Safety Authority classifies yersiniosis caused by *Y. enterocolitica* as one of the most important zoonotic diseases. The prevalence of *Y. enterocolitica* in cattle has not been thoroughly analyzed in Poland, and beef and bovine carcasses contaminated with antimicrobial resistant *Y. enterocolitica* pose a health risk for both, farm workers and consumers. Therefore, the aim of this study was to evaluate the prevalence of *Y. enterocolitica* in cattle and to determine the antimicrobial susceptibility of the isolated strains.

**Results:**

A total of 1020 samples were analyzed, including 660 rectal swabs collected from live cattle and 360 swabs from cold-stored beef carcasses. The results of this study indicate that *Y. enterocolitica* was isolated from three of the 15 examined cattle herds and the prevalence within these herds ranged from 0% to nearly 32%. *Y. enterocolitica* was isolated from 14.7% of the examined heifers, 7.4% of calves and 5.5% of adult cows. More than 65% of the strains were isolated from cold enrichment. The strains isolated from live cattle tested positive for the *ystB* gene, while *ail* and *ystA* genes were not found. Most of the isolated strains belonged to bioserotype 1A/NT. The majority of the isolated strains were resistant to ampicillin, cefalexin and amoxicillin with clavulanic acid, however these are expected phenotypes for *Y. enterocolitica.*

**Conclusions:**

The results of this study indicate that *Y. enterocolitica* is present in cattle herds in Poland. The strains isolated from live cattle were *ystB*-positive, most of them belonged to bioserotype 1A/NT. The prevalence of *Y. enterocolitica* strains was generally low in cold-stored beef carcasses.

## Background

*Yersinia enterocolitica* is a heterogeneous bacterial species that has been divided into six biotypes, based on their biochemical properties [1]. The strains belonging to biotypes 1B and 2–5 are regarded as pathogenic for humans and animals [2–4]. In turn, biotype 1A strains are classified as non-pathogenic [5, 6]. However, the presence of known and putative virulence associated features shared with pathogenic variants suggests a reassessment of the pathogenic potential of this biotype. [7]. *Y. enterocolitica* are divided into more than 70 serotypes based on somatic antigen O structure [8]. The optimal temperature for the growth of these bacteria is 22–29 C, but *Y. enterocolitica* can proliferate within a temperature range of 0 C to 45 C. However, at higher temperatures, the growth of *Y. enterocolitica* is usually inhibited by the accompanying flora [9, 10]. At a temperature of 4 C and low pH, *Y. enterocolitica* retains its pathogenic potential for several weeks [11], and its pathogenicity is maintained for up to several month in frozen food (around -18 C), which poses a significant health risk for consumers.

*Y. enterocolitica* is ubiquitous in the environment, and it easily colonizes various species of animals that become asymptomatic carriers that can act as potential sources of infection for humans. Many authors have reported a correlation between the strains isolated from pigs and clinical cases of humans yersiniosis, which suggests that pigs are the main reservoir of pathogenic *Y. enterocolitica* for humans and that the consumption of undercooked pork is an important source of infection [2, 3, 12].

*Y. enterocolitica* is a pathogen of growing epidemiological significance, and clinical cases of yersiniosis in humans have to be notified since 2002. Each year, the European Food Safety Authority (EFSA) classifies yersiniosis as one of the most important zoonotic diseases of the digestive tract. Yersiniosis was the third most commonly reported foodborne zoonotic disease in the EU in 2020 with a decreasing trend in 2016–2020 [13]. In humans, the most frequent clinical symptoms of yersiniosis are diarrhea, fever, abdominal pain, vomiting or blood in stools [3]. Small children are most frequently affected by yersiniosis, in older children and young adults, symptoms of disease may resemble those of appendicitis and may lead to hospitalization and potentially unnecessary appendectomies [14].

However, not all *Y. enterocolitica* are equally virulent*.*. The pathogenicity of *Y. enterocolitica* strains is determined mainly by the presence of virulence genes. The proteins encoded by these genes enable bacteria to penetrateinto susceptible individuals, colonize the digestive tract, evade the immune response, and grow under unfavorable conditions. One of the markers is the *ail* gene encoding the production of Attachment invasion locus (Ail) protein that enables *Y. enterocolitica* to become attached to and penetrate intestinal epithelial cells. Due to their low molecular weight (17 kDa), Ail proteins are easily masked by other surface structures such as lipopolysaccharide (LPS) [15]. In combination with *Yersinia* adhesin A (YadA), Ail protein confers cell binding, Yops (*Yersinia* outer proteins) delivery, and serum resistance activities [16]. The *ail* gene is an important virulence marker that is widely used to differentiate between pathogenic and non-pathogenic strains, as well as to detect foodborne pathogenic *Y. enterocolitica* [17–20].

One of the key attributes conditioning the pathogenicity of *Y. enterocolitica* strains is their ability to produce *Yersinia* stable toxin (Yst). Yst is a polypeptide chain containing 30 amino acids in C-terminal domain of a mature toxin and an N-terminal sequence of 18 amino acids which is trimmed during transport across the plasma membrane [21]. Two types of Yst enterotoxin have been identified to date: YstI (variants A, B and C) and the recently discovered and insufficiently investigated YstII [22]. *Y enterocolitica* strains isolated from cases of human yersinosis were able to produce YstI, suggesting that YstI type plays an important role in the etiology of diarrhea associated with yersiniosis.

There is evidence to indicate that various species of animals, including free-living animals, can be a significant reservoir of *Y. enterocolitica* [23–27]. However, the prevalence of *Y. enterocolitica* in cattle has not been thoroughly analyzed in Poland, and beef and bovine carcasses contaminated with *Y. enterocolitica* may pose a health risk for consumers. There are also reports that *Y. enterocolitica* strains may be multidrug resistant [1]. Therefore, the aim of this study was to evaluate the prevalence of *Y. enterocolitica* in cattle and to determine the bioserotype, molecular characteristics and antimicrobial susceptibility of the isolated strains.

## Results

### Isolation of *Y. enterocolitica* from herds and carcasses

**Fig. 1:**
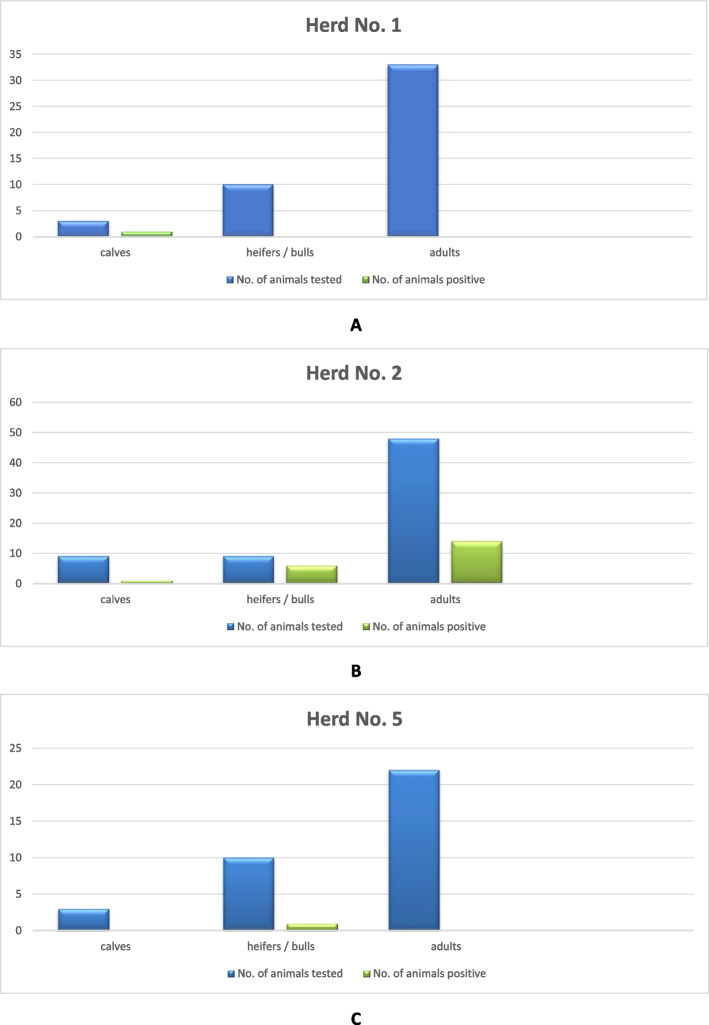
Isolation of *Y. enterocolitica* strains from cattle in herds No. 1, No. 2 and No. 5. The number of *Y. enterocolitica* strains isolated from different age groups of animals compared to the total number of animals in three herds where *Y. enterocolitica* was detected: A – herd No. 1; B – herd No. 2; C – herd No. 5

During the bacteriological analysis, *Y. enterocolitica* were isolated from rectal swabs collected from three of the 15 investigated cattle herds. In herd No. 1, two *Y. enterocolitica* strains were isolated from 92 samples. Both strains were isolated from the same animal (calf), but they were grown in two different types of culture. *Y. enterocolitica* was not identified in adult cattle, heifers or young bulls (Fig. 1).

In herd No. 2, *Y. enterocolitica* was identified in 26 of the 122 analyzed samples.Nine *Y. enterocolitica* strains were isolated from ITC enrichment, and 17 from cold enrichment.. In five animals (three adult cows and two heifers) two *Y. enterocolitica* strains grown in both ITC enrichment and cold enrichment were isolated. A positive result was noted in 21 of the 66 examined animals (31.8%). Strains were isolated from 14 of 48 adult cows, one of nine calves, and six of nine heifers (Fig. 1).

**Fig. 2:**
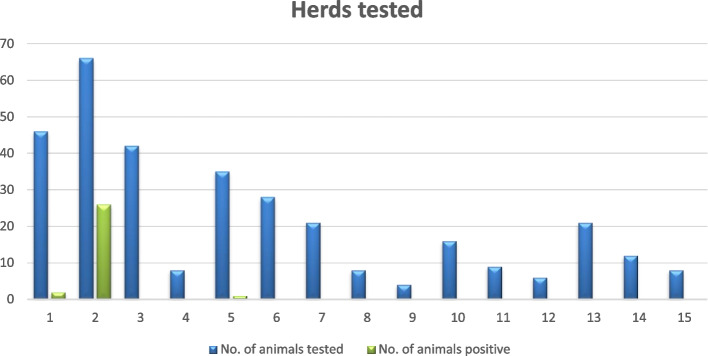
Isolation of *Y. enterocolitica* strains from cattle in the analyzed herds. The number of *Y. enterocolitica* strains isolated from animals belonging to the analyzed herds compared to the total number of animals in these herds

In herd No. 5, one *Y. enterocolitica* strain was isolated from 70 samples. The strain was grown in cold enrichment, and it was isolated from a sample collected from a heifer (Fig. 1). *Y. enterocolitica* was not detected in adult cattle or calves. In the remaining 12 herds, *Y. enterocolitica* was not isolated from ITC enrichment or cold enrichment with the use of standard bacteriological methods. The results of the bacteriological analyses of bovine rectal swabs are summarized in Fig. 2.

A total of 29 *Y. enterocolitica* strains were isolated from 23 animals. For six animals, two isolates were recovered from each animal, with one isolate from each enrichment approach*. Y. enterocolitica* was isolated from two of the 27 examined calves (7.4%), seven of 48 heifers (14.6%) and 14 of 255 adult cows (5.5%). Considerable differences were observed in the percentage of animals from which we isolated *Y. enterocolitica* to the total number of animals in one herd. The prevalence of *Y. enterocolitica* reached 32% in one herd, while in 12 herds it was 0%.

*Y. enterocolitica* was also isolated from 14 of the 180 examined beef carcasses, i.e. from 3.9% of the analyzed samples (two swabs were collected from each carcass). Three *Y. enterocolitica* strains were isolated from ITC enrichment with the use of standard bacteriological methods while cold enrichment produced positive results in 11 cases (Table 1).

**Table 1:** Results of bacteriological analyses and the culture media used for the isolation of *Y. enterocolitica* strains

Experimental group	Age group	Number of tested animals/carcasses	Number of *Y. enterocolitica*-positive animals/carcasses	Number of isolated *Y. enterocolitica* strains	Culture medium	
					**ITC**	**PSB**
**I**	calves	27	2	3	1	2
	heifers/young bulls	48	7	9	4	5
	adult cows	255	14	17	5	12
**II**	carcasses	180	14	14	3	11

In total, 43 *Y. enterocolitica* strains were isolated from 4.2% of all samples. Most strains (30 of 43, 69.8%) were isolated from cold enrichment, indicating that *Y. enterocolitica*, in particular those from beef carcasses, was isolated more effectively from cold enrichment than ITC enrichment.. The results of *Y. enterocolitica* isolation from ITC enrichment and cold enrichment are presented in Table 1.

### Bioserotyping analysis

All isolated strains were classified as biotype 1A due to their ability to ferment xylose, trehalose and esculin and produce pyrazinamidase, Twin esterase and indole. Biotypes 1B, 2, 3, 4 and 5 were not detected, and control strains were correctly identified. Serotyping of the 43 *Y. enterocolitica* revealed that 39 strains did not agglutinate with any of the applied reference sera; therefore, they were classified as NT. Four strains agglutinated with the O:5 antiserum. Strains belonging to serotype O:5 were isolated from both ITC enrichment (two strains) and cold enrichment (two strains).

One animal (calf from Herd 1) among the six animals from which two *Y. enterocolitica* strains were recovered from each animal, harbored two different serotypes, i.e. one NT and one O:5*.*

### Triplex PCR, HRM (High Resolution Melting) and sequencing

**Fig. 3:**
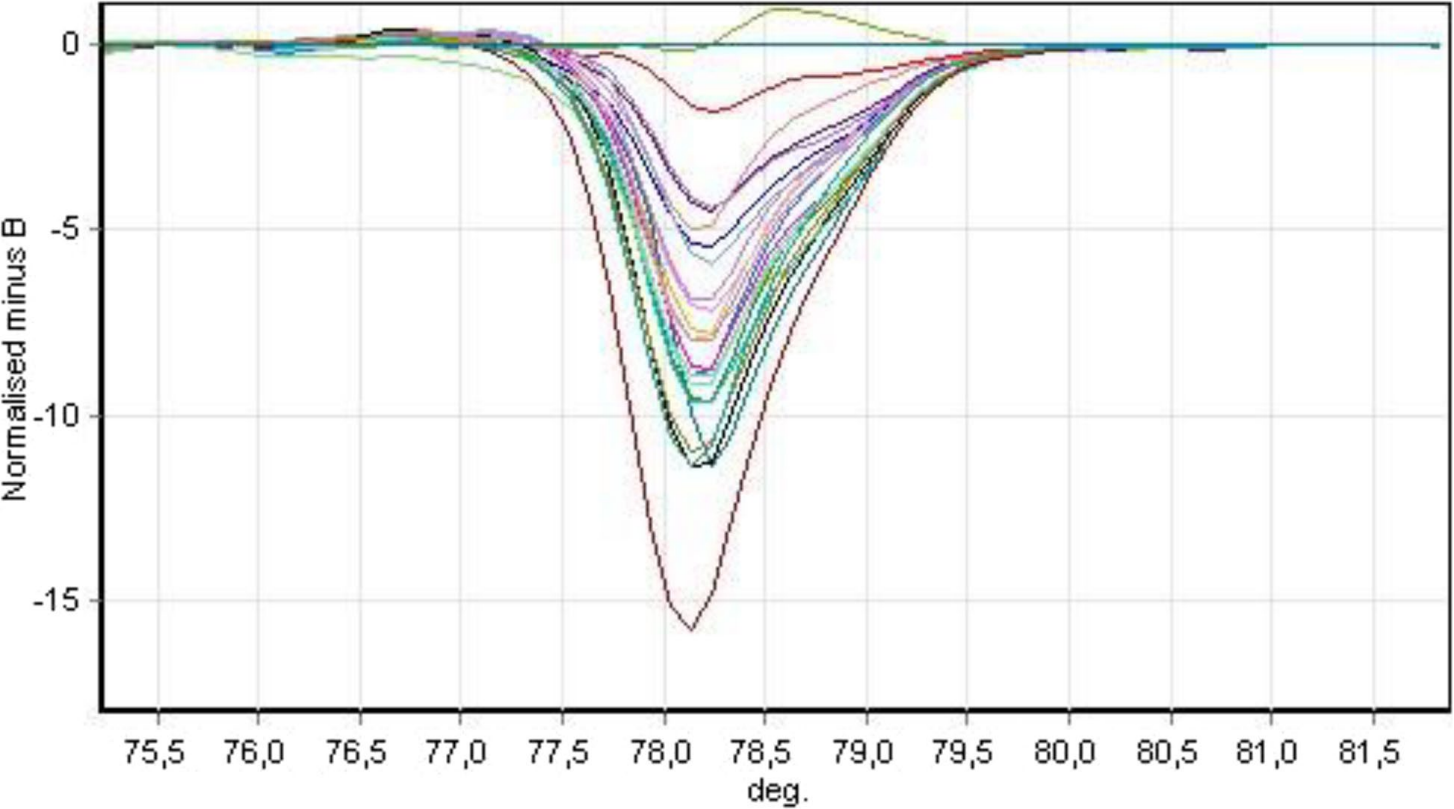
Analysis of SNPs of the *ystB* gene in *Y. enterocolitica* strains. Each line corresponds to a different nucleotide sequence of the *ystB* gene and is marked with a different color. The nucleotide sequences were compared with the reference sequences – the closer the lines, the greater the similarity between the compared sequences. Variations were defined as sequences not showing 100% homology with the reference sequences

The virulence markers of *Y. enterocolitica* were detected in triplex PCR with a primer set for the *ystA, ystB* and *ail* genes. All analyzed strains harbored *ystB* gene, which is consistent with the biotype and serotype of the isolated *Y. enterocolitica* strains. The reference strains were correctly identified with products of the correct size (Table 2). All *ystB*-positive strains were analyzed with the use of the HRM technique, and the results were processed in Rotor-Gene HRM software to classify the detected genes to the corresponding genotypes. An example of the result of the SNP (Single Nucleotide Polymorphism) analysis is presented in Fig. 3.

**Table 2:** Sequences of the primers used in triplex PCR

Gene	Primers sequences	Product size	Reference
*ail*	5’AATCACTACTGACTTCGGCTGG3’ 5’ACTATCTGAGATGATTAGAATCG3’	356 bp	Harnett et al., 1996 [28]
*ystA*	5’GTCTTCATTTGGAGGATTCGGC3’ 5’AATCACTACTGACTTCGGCTGG3’	134 bp	Platt-Samoraj et al., 2006 [29]
*ystB*	5’TGTCAGCATTTATTCTCAACT3’ 5’GCCGATAATGTATCATCAAG3’	180 bp	Platt-Samoraj et al., 2006 [29]

The SNP analysis of the *ystB* gene supported the identification of strains belonging to four genotypes (with 100% homology to the reference gene) and 12 variations (Table 3). Variations were defined as sequences not showing 100% homology with the reference sequences previously described by Bancerz-Kisiel et al. [30]. Thirteen sequences were classified as genotype 2; nine sequences as genotype 4; six sequences as genotype 3 and three sequences as genotype 1. The remaining 12 nucleotide sequences differed from the reference sequences and were defined as a variations. Direct sequencing of variations revealed nucleotide sequences with a length of 263 bp after trimming and folding, which is consistent with NCBI data. Three sequences were classified as genotype 1; one sequence was identified as genotype 2 and another one as genotype 3. The remaining seven variations did not contain sequences that were homologous with those published in the NCBI database. The greatest similarity was observed in the sequence of *Y. enterocolitica* strain 34 M PSB PL heat-stable enterotoxin type B* (ystB*) gene, partial cds, which was classified as variation 3 by Bancerz-Kisiel et al. [30] and deposited in NCBI under accession number KM253278. The identified strains were short of one C118T mutation relative to the above nucleotide sequence. Therefore, the study revealed new variants of the nucleotide sequence of the *ystB* gene which are published in NCBI under accession numbers OR113048 – OR113054.

**Table 3:** Results of the HRM analysis

Genotype	Reference sequence		No. of tested *Y. enterocolitica*
	**NCBI No**	**Description**	
1	D88145.1	*Y. enterocolitica* DNA for *Yersinia* Heat-stable Enterotoxin Type B, complete cds	3
2	KM253283	*Y. enterocolitica* strain 156 PSB PL heat-stable enterotoxin type B* (ystB*) gene	13
3	KJ592626	*Y. enterocolitica* strain 237 PSB PL heat-stable enterotoxin type B* (ystB*) gene	6
4	KU198401	*Y. enterocolitica* strain KA16 PSB PL heat-stable enterotoxin type B* (ystB*) gene	9
Variation	-	sequence without 100% homology with the used reference sequences	12

**Fig. 4:**
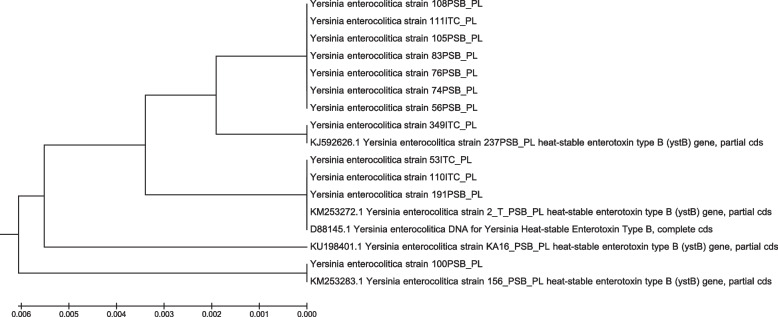
Phylogenetic analysis of *Y. enterocolitica* strains isolated from cattle. The evolutionary history was inferred using the UPGMA method. The tree is drawn to scale, with branch lengths in the same units as those of the evolutionary distances used to infer the phylogenetic tree. The evolutionary distances were computed using the Maximum Composite Likelihood method and are in the units of the number of base substitutions per site. The analysis involved 17 nucleotide sequences. Codon positions included were 1st + 2nd + 3rd + Noncoding. All positions containing gaps and missing data were eliminated. There were a total of 253 positions in the final dataset. Evolutionary analyses were conducted in MEGA5

The strains isolated from cattle were subjected to a phylogenetic analysis based on partial nucleotide sequences of the *ystB* gene, which revealed five phylogenetic groups of strains. Four groups consisted of regular genotypes that were described above, whereas the fifth group was composed of variations with previously unidentified nucleotide sequences of the *ystB* gene (Fig. 4). Genotype 2 (14 strains) and variations (seven strains) were predominant in the strains collected from live cattle, whereas the remaining genotypes were less frequently represented. In the six animals from which two *Y. enterocolitica* strains were obtained, differences in genotype were observed in two cases. Genotype 2 and genotype 3 were identified in two *Y. enterocolitica* strains from one calf (Herd 1), while genotype 4 and variation were identified in two *Y. enterocolitica* strains from one heifer (Herd 2). In general, the strains isolated from heifers and young bulls in herd No. 2 were characterized by the greatest variation (genotype 1, genotype 4 and variations). Most of the strains isolated from adult cattle in herd No. 2 belonged to genotype 2 (12 strains). Four variations and genotype 1 (one strain) were also noted, and a strain belonging to genotype 2 was isolated from a calf in this herd. The strain isolated from a heifer in herd No. 5 was classified as genotype 1. Most of the strains isolated from cold-stored carcasses belonged to genotype 4 (six strains), genotype 3 (five strains) and genotype 1 (three strains). Variations were not detected.

### Antimicrobial susceptibility analysis

**Fig. 5:**
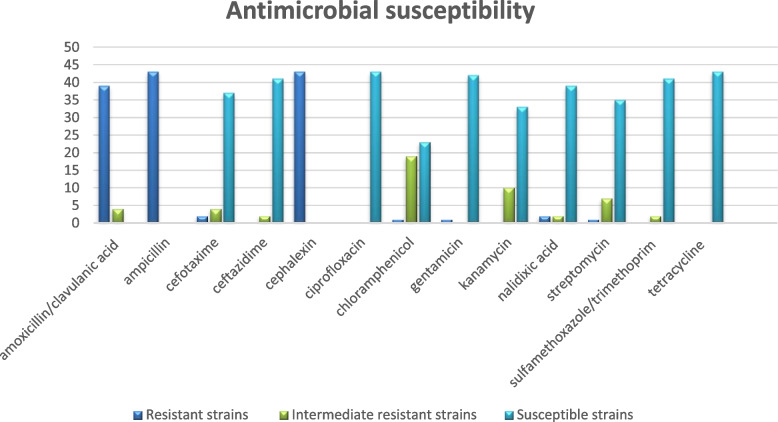
Antimicrobial susceptibility of *Y. enterocolitica* strains isolated from cattle. The number of *Y. enterocolitica* strains resistant/intermediate resistant/susceptible to 13 examined antimicrobials

Susceptibility to antimicrobials was determined in molecularly confirmed *Y. enterocolitica* strains isolated from live cattle and cold-stored beef carcasses with the use of the standardized disc diffusion method. Susceptibility to 13 antimicrobials was tested. All strains were resistant to ampicillin and cefalexin. The majority of the examined strains were also resistant to amoxicillin with clavulanic acid, and only four strains were intermediate resistant to this antibiotic. All strains were susceptible to ciprofloxacin and tetracycline, and most strains were also susceptible to ceftazidime (two intermediate resistant strains), sulfamethoxazole/trimethoprim (two intermediate resistant strains) and gentamicin (one resistant strain). The examined *Y. enterocolitica* strains were characterized by varied susceptibility to the remaining antimicrobials: cefotaxime, chloramphenicol, kanamycin, nalidixic acid and streptomycin (Fig. 5).

No differences in antimicrobial susceptibility were observed between *Y. enterocolitica* strains isolated from live cattle and carcasses, or between the analyzed herds. In five of the six animals, where two strains grown in different types of culture were isolated from each animal, the detected strains varied in susceptibility to the tested antimicrobials. Four pairs of these strains responded differently to two antimicrobials: amoxicillin with clavulanic acid and chloramphenicol in two animals; cefotaxime and kanamycin in one animal; cefotaxime and chloramphenicol in one animal. Two strains varied in susceptibility to five antimicrobials: cefotaxime, chloramphenicol, kanamycin, nalidixic acid and streptomycin. In case of cefotaxime and streptomycin, one strain in the pair was resistant and the other was susceptible. The vast majority of strains (76.74%) were resistant to three antimicrobials, six strains (13.95%) were resistant to four antimicrobials, and four strains (9.31%) were resistant to two antimicrobials. It means that 100% strains were resistant to more than one antimicrobial agent, and 39 (90.69%) displayed resistance to three or more antimicrobials. However, these results are completely different if we exclude from the analysis (according to the EUCAST guidelines for strains isolated from humans [31]) the common occurrence of *Y. enterocolitica* resistance to ampicillin, amoxicillin with clavulanic acid as well as first generation cephalosporins such a cefalexin. Considering the above guidelines, only seven strains showed resistance to one of the tested antimicrobials: two strains to cefotaxime, two strains to nalidixic acid, one strain to chloramphenicol, one strain to gentamicin and one strain to streptomycin.

## Discussion

In the present study, *Y. enterocolitica* strains were isolated from three of the 15 examined cattle herds. A similar study was carried out by Schmid et al. [32] in southern Bavaria, Germany. They collected samples from 49 cattle herds, including 34 dairy cattle herds (beef cattle were present in 30 herds) and 15 beef cattle herds, and reported positive results in three dairy cattle herds (8.8%) and one beef cattle herd (6.7%). In total, *Y. enterocolitica* was isolated from four of the 49 herds (8.2%), which suggests that this pathogen is less prevalent in Bavarian than in Polish cattle (20%). It should be noted that in the current study, the prevalence of *Y. enterocolitica* differed considerably between the examined herds, ranging from not detected (12 herds) to nearly 32% (one herd).

Schmid et al. [32] isolated a total of six *Y. enterocolitica* strains (1.6%) from 382 cattle fecal samples. In the present study, 29 *Y. enterocolitica* strains were isolated from 660 samples collected from cattle, which accounts for 4.4% of all samples. Similar results were reported by Milnes et al. [33, 34] who examined rectal samples collected from cattle in the United Kingdom. *Y. enterocolitica* was isolated from 30 (4.5%) of the 672 analyzed samples, and its prevalence in cattle was considerably influenced by season. The risk of infection was higher between December and May, which is consistent with previous reports [35] and corresponds with the psychrophilic properties of *Y. enterocolitica.* In the present study, samples were collected in a similar period to that described by Milnes et al. [34]. Milnes et al. [34] found that that in addition to season, the prevalence of *Y. enterocolitica* was also affected by the animal’s age and was higher in older individuals. The present study where *Y. enterocolitica* was more frequently from younger animals (heifers and calves) are in contrast to the observations above.

More than 65% of the *Y. enterocolitica* obtained in the current study were isolated after cold enrichment, which suggests that both enrichments should be used to produce reliable data. *Y. enterocolitica* strains from cold-stored carcasses were even more effectively isolated from cold enrichment. Fourteen strains were isolated from carcasses, of which 11 were isolated from cold enrichment and only three from ITC enrichment. To the best of the author’s knowledge, the prevalence of *Y. enterocolitica* in cold-stored beef carcasses has never been investigated to date; therefore, the present findings could not be directly compared with published data. In previous studies of carcasses of game animals, including free-living ruminants [36], *Y. enterocolitica* were isolated from 60% roe deer carcasses, 43.8% of red deer carcasses and 55% wild boar carcasses. The prevalence of *Y. enterocolitica* was much higher in game animals than in cattle probably because game carcasses are not skinned before refrigeration, which promotes meat aging but, as evidenced by the cited study, compromises the microbiological safety of meat. To compare, the prevalence of *Y. enterocolitica* in pig carcasses, previously studied by other authors, varies greatly. *Y. enterocolitica* was recovered from 39.7% of carcass surfaces post-evisceration by Van Damme et al. [37] in Belgium. However, in the study of Powell et al. [38], conducted in the United Kingdom, the prevalence of *Y. enterocolitica* in carcasses was only 9.6%, which was significantly lower than the previous survey.

Ye et al. [39] screened 2320 food samples, including 76 samples of beef, for the presence of *Y. enterocolitica* between 2011 and 2014. Five strains (10.6%) were isolated from 47 beef samples collected in summer, and five strains (17.2%) were isolated from 29 samples obtained in winter. This was the first study to provide information about food contamination with *Y. enterocolitica* in China, and it confirmed that the pathogen’s prevalence varies across seasons. In a study conducted by Kilonzo-Nthenge et al. [40] in the United States, the prevalence of *Y. enterocolitica* in 24 raw beef samples was also low at 4.2%. *Y. enterocolitica* was more frequently isolated from beef in the work of Mayrhofer et al. [41] who evaluated the antimicrobial resistance profiles of the five major food-borne pathogens in Austria and isolated *Y. enterocolitica* from 29 (31.9%) of the 91 examined beef samples. Research studies investigating the presence of *Y. enterocolitica* in cow’s milk also produced interesting findings. Bernardino-Varo et al. [42] analyzed 1300 samples of raw milk and isolated *Y. enterocolitica* from 454 samples.. *Y. enterocolitica* accounted for 44.3% of all strains belonging to the genus *Yersinia*. The prevalence, pathogenic potential and antimicrobial resistance of *Yersinia* spp. in raw cow's milk in Iran was evaluated by Jamali et al. [43] in 2008–2010. They examined 240 samples of milk and isolated *Y. enterocolitica* from 14 samples (5.8%).

The *Y. enterocolitica* isolated in the present study belonged to biotype 1A. This biotype is generally considered non-pathogenic, but according to some authors, its pathogenic potential cannot be excluded [7]. McNally et al. [44] argued that biotype 1A strains are the predominant etiological factor of yersiniosis in the Commonwealth countries. According to Huovinen et al. [3] the majority of *Y. enterocolitica* strains isolated from Finnish patients belong to biotype 1A. In a study from the United Kingdom, the *Y. enterocolitica* isolated from cattle belonged to biotype 1A [33]. Similar to our study, most of the *Y. enterocolitica* strains were non-typable by serotyping*.*

Biotype 1A strains were also predominant in a study of cattle conducted by McNally et al. [45] in Scotland, England and Wales. In the group of 56 isolated strains, only two (4.4%) belonged to biotype 3 (serotype O:5,27), whereas the remaining strains were classified as biotype 1A. Fourteen strains (30.4%) were serologically non-typable, and similar results were noted in the present study and in the work of Milnes et al. [33]. These findings indicate that biotype 1A strains are far more diverse than strains of any other biotype of *Y. enterocolitica*. Biotype 1A predominated also in *Y. enterocolitica* strains isolated from cold-stored carcasses of free-living animals [36], strains isolated from beef samples [39] and cow’s milk [42].

In the current study, all isolated strains harbored *ystB* gene, which is consistent with the results of biotype and serotype analyses. None of the strains contained *ail* and *ystA* genes. Schmid et al. [32] isolated six *Y. enterocolitica* strains from 382 samples of bovine feces in Bavaria and conducted real-time PCR to detect the *ail* gene, which was subsequently detected in six *Y. enterocolitica* isolates. In a study of *Y. enterocolitica* isolated from raw milk, Jamali et al. [43] detected the *ail* gene in only one of 14 *Y. enterocolitica* strains.. This strain belonged to bioserotype 4/O:3, and it also harbored the *ystA* gene. The *ystA* gene were reported in all strains belonging to bioserotype 1B/O:8, whereas biotype 1A strains harbored *ystB* gene [43]. In turn, Bancerz-Kisiel et al. [36] identified *ystB* gene in all *Y. enterocolitica* strains isolated from refrigerated carcasses of hunter-harvested game animals, including free-living ruminants. Ye et al. [39] also searched for the *ail*, *ystA* and *ystB* genes in food products in China to distinguish between pathogenic and non-pathogenic *Y. enterocolitica* strains. The *ail, virF*, *ystA* and *ystC* genes were not identified in 10 *Y. enterocolitica* biotype 1A strains isolated from raw beef. However, the analyzed strains harbored *ystB, fepD, ymoA, fes* and *sat* genes. The *ystB* gene was regarded as the most important virulence marker in biotype 1A strains, which corroborates the findings of other authors.

It should be mentioned that different molecular typing techniques have been developed to more accurately describe *Y.* *enterocolitica* strains [46]. Development of sequence-based phylogenetic methods, including HRM, but also the highly reproducible and portable Multilocus Sequence Typing (MLST) (reported in EnteroBase) allow identification of *Yersinia* isolates at the species and infra-specific levels [47]. Comparison of the *Y. enterocolitica* isolates is also done using Pulsed-Field Gel Electrophoresis (PFGE) and Multi-locus variable number tandem repeat analysis (MLVA) [46]. In the present study, the isolated strains formed five phylogenetic groups. In the pool of strains isolated from live cattle, genotype 2 was predominant, and the remaining genotypes were detected far less frequently. The results of the present study can be compared with the work of Bancerz-Kisiel et al. [30] who analyzed SNPs in the *ystB* gene of *Y. enterocolitica* strains isolated from various species of free-living animals. Genotype 2 dominated in *Y. enterocolitica* strains isolated from roe deer immediately after harvest. Many *Y. enterocolitica* strains isolated from refrigerated roe deer carcasses also belonged to genotype 2, but phylogenetic diversity was much higher in this group of strains [30]. Similar diversity was observed in the strains isolated from cold-stored beef carcasses in the present study. Genotypes 4 and 3 were most prevalent, and in the work of Bancerz-Kisiel et al. [30], these genotypes were most often detected in red deer and wild boar carcasses, respectively, immediately after harvest. In the present study, the fifth phylogenetic group was particularly interesting because it contained variations whose nucleotide sequence of the *ystB* gene has not been observed previously. These strains were isolated only from live animals, and the above variations were not noted in *Y. enterocolitica* strains isolated from cold-stored carcasses.

In the last stage of the study, *Y. enterocolitica* strains were analyzed for susceptibility to antimicrobials with the use of the standardized disc diffusion method. The isolated strains were susceptible to ciprofloxacin and tetracycline, and most strains were also susceptible to ceftazidime, sulfamethoxazole/trimethoprim, and gentamicin. The analyzed strains differed in susceptibility to the remaining antimicrobials. Some strains were resistant to multiple antimicrobials: more than 90% of the isolated strains were resistant to at least three antimicrobials. However, these results were mainly obtain based on testing of ampicillin, amoxicillin with clavulanic acid and cephalexin. According to the EUCAST guidelines [31] these are expected phenotypes, and therefore strains could not be described as multidrug resistant.

The same antimicrobials were used by Ye et al. [39] to test the susceptibility of 10 *Y. enterocolitica* strains isolated from raw beef. Sulfonamide, imipenem and ticarcillin discs were also used in their experiment. All *Y. enterocolitica* strains were susceptible to kanamycin and sulfonamides, but they were also resistant to three or more antimicrobials. Most of the examined strains were resistant to ampicillin and cefalexin (90%), sulfamethoxazole/trimethoprim (70%) and amoxicillin with clavulanic acid (40%). All strains harbored the genes encoding the production of β-lactamases. The results reported by Ye et al. [39] are consistent with the observations made in the present study. In both studies, the highest number of strains were resistant to ampicillin, first-generation cephalosporins, and amoxicillin with clavulanic acid. The greatest differences were noted between resistance to sulfamethoxazole/trimethoprim. In the current study, most strains were susceptible to this antimicrobial, whereas the strains isolated by Yet et al. [39] were largely resistant. Mayrhofer et al. [41] analyzed the resistance of 29 *Y. enterocolitica* strains isolated from beef samples to tetracycline, gentamicin, kanamycin, sulfonamide, nalidixic acid, trimethoprim, chloramphenicol and streptomycin. All strains, including 26 biotype 1A strains, were susceptible to the tested antimicrobials. Jamali et al. [43] examined the antimicrobial susceptibility of *Y. enterocolitica* strains isolated from cow’s milk, but did not present separate results for the strains isolated from raw cow’s milk and the strains isolated from raw goat’s and sheep’s milk. A total of 19 *Y. enterocolitica* strains were analyzed, 14 of which were isolated from cow’s milk; 52.6% of these strains were resistant to tetracycline, and 26.3% were resistant to ciprofloxacin. Only 15.8% of the isolated strains were resistant to ampicillin and first-generation cephalosporins.

Special attention should be paid to strains that were isolated from the same animal, but were grown in two different types of culture. These strains often differed in properties, serotype, genotype and antimicrobial susceptibility. A difference in serotype was observed in two strains isolated from a calf in herd No. 1. One of the strains was serologically non-typable, whereas the other belonged to serotype O:5. Differences in genotype were observed in two cases: the above-mentioned calf in herd No. 1 (where one of the isolated strains belonged to genotype 2 and the other belonged to genotype 3) and a heifer in herd No. 2 (genotype 4 and a variation). In five of six cases where two strains were isolated from the same animal, the strains in each pair differed in susceptibility to antimicrobials. In four cases, differences were noted in susceptibility to two antimicrobials, and in one case, the strains differed in susceptibility to four antimicrobials, including cefotaxime and streptomycin, where one strain in the pair was susceptible and the other was resistant. The strains isolated from the calf in herd No. 1 were also characterized by varied antimicrobial susceptibility, which indicates that they differed in serotype, genotype as well as resistance to the tested antimicrobials. Bancerz-Kisiel et al. [36] also isolated strains of different biotypes and serotypes from cold-stored carcasses of game animals, and attributed these differences to mixed infections.

## Conclusion

The prevalence of *Y. enterocolitica* in live cattle and cold-stored bovine carcasses has not been studied in Poland to date. Therefore, the present study provides valuable information for consumers, in particular in Poland, where beef is frequently served raw or undercooked. The results of the present study indicate that *Y. enterocolitica* is present in cattle herds in Poland, but its prevalence varies considerably. The strains isolated from live cattle tested positive for the *ystB* gene, and most of them belonged to bioserotype 1A/NT. The vast majority of the isolated strains were resistant to ampicillin, cefalexin and amoxicillin with clavulanic acid, however these are expected phenotypes for *Y. enterocolitica*. The prevalence of *Y. enterocolitica* strains was generally low in cold-stored beef carcasses.

## Methods

### Materials

Two groups of swabs were used in the analysis: I – rectal swabs collected from live cattle showing no symptoms of yersiniosis, and II – swabs from beef carcasses that were cold-stored in an abattoir. Rectal swabs were obtained during clinical veterinary examinations from 330 animals from 15 herds (differed in size, productivity and hygiene status) between January and April 2018. All animals within the herds were tested. Sample collection was performed according to the Act for the Protection of Animals for Scientific or Educational Purposes of 15 January 2015 (Official Gazette 2015, No. 266), applicable in the Republic of Poland. Since the samples were collected during veterinary procedures these activities did not require additional agreement from the Bioethical Committee. Informed consent was obtained from animal breeders for sampling. Carcass swabs were collected from 180 beef carcasses that were cold-stored in an abattoir for minimum 48 h. Swabs were obtained from the region of the tenderloin, a primal cut of beef that can be consumed raw (tartar steak) or undercooked (rare and medium rare steak). Two swabs were collected from each animal and carcass. A total of 1020 swabs were acquired, including 660 rectal swabs collected from live cattle and 360 swabs from cold-stored beef carcasses. All methods used in the present study were carried out in accordance with relevant guidelines and regulations.

### Isolation of *Y. enterocolitica* from herds and carcasses

Detection and isolation of *Y. enterocolitica* was performed using ISO 10273:2017 [48] with modification of cold enrichment extended to three weeks. Swabs were collected with Amies media swabs (Deltalab) and enriched in two types of liquid media: 9 ml of irgasan, ticarcillin and potassium chlorate (ITC, Biocorp Ltd, Poland) – ITC enrichment, and broth with peptone, sorbitol, and 9 ml of bile salts (PSB Biocorp Ltd, Poland) – cold enrichment. The ITC were incubated at a temperature of 25 °C for 48 h, and samples cultured in PBS were incubated at a temperature of 4 °C for 3 weeks. After incubation, 0.5 ml of the enriched culture was transferred to 4.5 ml of 0.5% KOH solution and shaken for 20 s. A loopful (0.01 ml) of the KOH treated enrichment broth was plated on *Yersinia* selective agar (CIN – Cefsulodin-Irgasan-Novobiocin, Merck KGaA, Germany) to obtain single colonies. The plates were incubated at 30 °C for 24 h, and they were evaluated under a magnifying glass with a light source. When cultured on CIN agar, *Y. enterocolitica* forms small colonies with an estimated diameter of 1 mm, smooth and semi-transparent borders, and a dark red, non-opalescent center. Plates that did not contain characteristic *Y. enterocolitica* colonies or where colony growth was slow or non-specific were left in the incubator for another 24 h. Five typical colonies from each plate were selected for further analysis.

### Bioserotyping analysis

The biotype of the isolated *Y. enterocolitica* strains was determined with the use of the protocol described in Annex D of standard PN-EN ISO 10273:2017 [48]. *Y. enterocolitica* strains were biotyped based on their ability to ferment trehalose, xylose and esculin, and produce pyrazinamidase, Tween esterase and indole, and serotyped by slide agglutination using antisera for O:3,O:5,O:8, O:9 and O27 (Sifin Diagnostics, Germany). Twenty-four hour colonies cultured on blood agar were suspended in a drop of 0.85% NaCl on a microscope slide. A drop of serum was applied to the slide and combined with the colony with the use of an inoculation loop, and the mixture was shaken for 1 min. The result of the test was positive if agglutination occurred with one of the five tested sera. Strains that did not agglutinate with any of the sera were regarded as non-typable (NT).

### Triplex PCR, HRM and sequencing

Genomic DNA was isolated with the Genomic Mini kit (A&A Biotechnology, Gdynia) for isolating genomic DNA from bacteria, cell cultures and solid tissues. DNA was isolated according to the manufacturer’s instructions. Molecular analysis involved triplex PCR with the amplification of the fragments of *ail*, *ystA* and *ystB* genes. The sequences of the primers synthesized by Genomed (Warsaw, Poland) were previously described by Harnett et al. *(ail*) [28] and Platt-Samoraj et al. (*ystA, ystB)* [29] (Table 2).

Triplex PCR was conducted with the HotStarTaq *Plus* DNA Polymerase Kit (Qiagen) and the HotStarTaq *Plus* Master Mix Kit (Qiagen) according to the procedure described by Bancerz-Kisiel et al. [24]. The reaction mix with a total volume of 20 µl contained around 120 ng of isolated DNA (1 to 3 µl), 10 µl of the HotStarTaq Plus Master Mix 2x, 2 µl of CoralLoad Concentrate 10x, 0.1 µl of each primer (with a final concentration of 0.5 µM), supplemented to 20 µl with RNase-Free Water. Three controls were used for each PCR assay: two positive controls with DNA isolated from the reference strains (strain ACTT 23715, biotype 1B, serotype O:8; and a reference strain for biotype O:5), and one negative control without DNA. Triplex PCR was performed in the Mastercycler thermal cycler (Eppendorf) under the following conditions: initial denaturation at 95 °C for 5 min, followed by 30 cycles of denaturation at 94 °C for 30 s, primer annealing at 54 °C for 30 s, amplification at 72 °C for 1 min, and final chain synthesis at 72 °C for 10 min. The PCR products were separated by gel electrophoresis (2% agarose), visualized using the GelDoc System (Bio-Rad) and amplicon size determined by comparison with a DNA standard (GeneRuler™ 100 bp, Ladder Plus, Fermentas).

Single nucleotide polymorphisms (SNPs) of the *ystB* gene were identified and analyzed by the High Resolution Melting (HRM) technique using the Rotor-Gene 6000™ real-time cycler (Qiagen) with a dedicated HRM channel. Mutations were identified by analyzing the melting curves of amplification products during each experiment. Novel primer sequences for *ystB-1* (5`GGACACCGCACAGCTTATATTTT3`) and *ystB-2* (5`GCACAGGCAGGATTGCAACA3`) [2] were used. The obtained amplicons had a size of 319 bp, which supported an analysis of a longer sequence. The reaction was carried out under the following conditions: initial denaturation at 95 °C for 5 min, followed by 40 two-stage cycles, where the first stage was conducted at 95 °C for 10 s, and the second stage was conducted at 46 °C for 30 s. The HRM analysis was conducted every 0.1 °C in the temperature range of 65–90 °C, with a time hold of 2 s. After the second stage of each cycle, fluorescence was measured in the HRM channel. The reaction was conducted using the Type-it HRM PCR Kit (containing HotStarTaq Plus DNA Polymerase, EvaGreen intercaling dye, Q-Solution, dNTPs and MgCl_2_). The results were analyzed with the use of Rotor-Gene HRM software, and amplicons were classified to genotypes by comparison with the previously published standards [30]. The specificity of randomly selected amplicons was confirmed by direct sequencing. Selected amplicons were purified with the CleanUp kit (A&A Biotechnology, Gdynia) according to the manufacturer’s instructions and sequenced by Genomed (Warsaw, Poland). The obtained sequences were assembled with the Lasergene v 8.1.5 software (DNASTAR, Madison, WI, USA) and compared with the previously described nucleotide sequences with the use of the ClustalW algorithm [49] in the Mega 5.2.1 program [49]. The nucleotides and sequences were identified with BioEdit v. 7.2.0 application. The phylogenetic tree was generated in the Mega 5 program [50].

### Antimicrobial susceptibility analysis

The antimicrobial susceptibility of molecularly confirmed *Y. enterocolitica* strains was determined by the standardized disc diffusion method on Mueller–Hinton agar (Oxoid, Thermo Scientific) after 24 h of incubation at a temperature of 30 °C. The resistance to antimicrobials was assessed according to the CLSI (Clinical and Laboratory Standards Institute, USA) [51] guidelines using the quality control strain *Escherichia coli* ATCC 25922. Thirteen different discs were used in the analysis (Oxoid, Thermo Scientific): amoxicillin + clavulanic acid (20/10 µg), ampicillin (10 µg), cefotaxime (30 µg), ceftazidime (30 µg), cephalexin (30 µg), ciprofloxacin (5 µg), chloramphenicol (30 µg), gentamicin (10 µg), kanamycin (30 µg), nalidixic acid (30 µg), streptomycin (10 µg), sulfamethoxazole/trimethoprim 19:1 (23.75/1.25 µg), and tetracycline (30 µg). The results were interpreted by measuring the diameter of the zones of complete inhibition, including the diameter of the disc. Then, the measurement result was assigned to the appropriate category.

## Data Availability

The datasets used and/or analysed during the current study will be made available from the corresponding author on reasonable request. New variants of the nucleotide sequence of the *ystB* gene are published in NCBI under accession numbers OR113048 – OR113054.

## Abbreviations

*Y. enterocolitica*: Yersinia enterocolitica
Ail: Attachment invasion locus
LPS: Lipopolysaccharide
YadA: *Yersinia* Adhesin
Yst: *Yersinia* Stable toxins
ITC: Irgasan, ticarcillin and potassium chlorate
PSB: Broth with peptone, sorbitol, and bile salts
NT: Non-typable
SNPs: Single nucleotide polymorphisms
HRM: High-Resolution Melting
CLSI: Clinical and Laboratory Standards Institute
