# Correction: Isolation, characterization and antimicrobial resistance of *Yersinia enterocolitica* from Polish cattle and their carcasses

**DOI:** 10.1186/s12917-023-03734-w

**Published:** 2023-09-22

**Authors:** Piotr Łada, Klaudia Kończyk‑Kmiecik, Agata Bancerz‑Kisiel

**Affiliations:** Department of Epizootiology, Faculty of Veterinary Medicine, University of Warmia and Mazury, Oczapowskiego 2 St, 10‑719, Olsztyn, Poland

Correction: *BMC Vet Res* **19**, 143 (2023)

10.1186/s12917-023-03700-6.

Following publication of the original article [1], the authors would like to correct the texts under the Funding section.

The texts currently read:

Publication costs financed by the Minister of Science and Higher Education in the rangę of the program entitled “Regional lnitiative of Excellence” for the years 2019–2023, Project No. 010/RID/2018/19, amount of funding 12.000.000 PLN.

The texts should read:

Publication costs financed by the Minister of Education and Science under the program entitled “Regional Initiative of Excellence” for the years 2019–2023, Project No. 010/RID/2018/19, amount of funding 12.000.000 PLN.
